# A comparison of the effects of teaching through simulation and the traditional method on nursing students' self-efficacy skills and clinical performance: a quasi-experimental study

**DOI:** 10.1186/s12912-022-01065-z

**Published:** 2022-10-20

**Authors:** Marzieh Azizi, Ghobad Ramezani, Elham Karimi, Ali Asghar Hayat, Seyed Aliakbar Faghihi, Mohammad Hasan Keshavarzi

**Affiliations:** 1 Clinical Education Research Center, School of Medicine, Shiraz University of Medical Sciences, Shiraz, Iran; 2 Center for Educational Research in Medical Sciences (CERMS), Department of Medical Education, School of Medicine, Iran University of Medical Sciences (IUMS), Tehran, Iran

**Keywords:** Simulation, Self-efficacy skills, Clinical performance, Students of nursing

## Abstract

**Introduction:**

Simulators in a clinical environment provide a space where students can acquire skills and experience under the supervision of their professors without any worries or inflicting any harm on their patients. The current study aimed to compare the effects of teaching through simulation and the traditional method on nursing students' self-efficacy skills and clinical performance.

**Method:**

The current study was quasi-experimental and adopted a pre-test & post-test design. The population consisted of 122 students of nursing, out of whom 100 students were selected as the sample. Then, they were randomly divided into an experimental and a control group. A questionnaire assessed the students' self-efficacy skills and clinical performance before and after implementing the instructional programs. The data were analyzed using descriptive and inferential statistical techniques in SPSS 23.

**Findings:**

The mean of the participants' self-efficacy scores increased significantly after the intervention (from 87.57 to 142.13). Moreover, the mean of the participants' clinical performance increased significantly after the intervention (from 2.16 to 4.57). The findings indicated that simulation teaching significantly affects nursing students' self-efficacy and clinical performance.

**Conclusion:**

Simulation was recommended as an effective teaching methodology, particularly in nurses' internship wards. In other words, acquiring the essential skills through applying the simulation method is recommended before entering real-world environments.

## Introduction

Simulation is a version of some actual instruments or working conditions designed to illustrate some behavioral dimensions of a physical or abstract system via the behavior of another system. The main goal of simulation in healthcare is to prepare students to face clinical situations, increase patients' safety, reduce errors, and improve nurses' clinical judgments. Thus, students can get a command of the skills that are less likely to be acquired in real-world situations [1]. Nurses need constant learning to update their knowledge and skills [2]. In other words, medical knowledge is increasingly being expanded. Thus, teaching nursing should become a dynamic form of teaching and move towards innovation, advancement, and implementation of modern teaching and educational methods [3]. Nowadays, IT-based instruments have significantly permeated all areas of medical sciences, particularly education [4]. Continuous education by applying technologies can bring essential advantages such as enhancing knowledge and effectiveness, improving performance quality, and increasing nurses' skills and professional qualifications [1].

Moreover, the method can bring about flexibility, easy access, and an opportunity to play valuable roles in nursing education at any time [5]. Simulation-based nursing education is a pedagogical approach that is increasingly gaining popularity. Simulation as a teaching method includes activities that mock an actual clinical environment and has been designed to illustrate processes, decision-making, and critical thinking via techniques such as role-play or instruments like educational films and models, scenarios, and case studies [6, 7]. The use of simulation in nursing education seems justifiable due to the lack of sufficient feedback in clinical environments, patients' inactivity during the examination process, the lack of access to a sufficient number of patients, the ever-changing composition of patients in real-world conditions, the lack of available clinical situations, and the significant number of students in clinical environments [8]. Simulation can improve students' knowledge, skills, and performance in nursing education. Students attain higher levels of critical thinking by practicing and acquiring new professional skills without interfering with patients' safety and health [9]. Implementing the method can also enhance nurses' learning and provide helpful feedback so they can become skillful nurses. The simulation scenarios empower nursing students in applying the educational, perceptual, and psychological experiences of learning and help students to acquire the skills necessary for thinking, evaluating, problem-solving, decision making, and analyzing the data [10]. Transferring the information concerning classrooms and clinical environments to the simulated environment in a different way and portraying the characteristics of clinical situations and people's actual lives are some features of simulation-based education [11]. The method can increase the tendency to group work and expand the spirit of cooperation and collective influence among the students. Simulated environments can provide places for the students to expand their capabilities in terms of the specialized skills of their professions without imposing any threat to the patients' lives [12].

Nursing students play a key role in continuously taking care and improving patients' health and making the healthcare system more efficient in the future. Thus, enhancing the quality of the healthcare system is a major concern, and nurses' performance is an important and significant factor that influences healthcare quality [13]. On the other hand, efficient nurses need skills like problem-solving and capabilities such as making decisions for clinical situations. Therefore, clinical performance is important in the nursing education system [14]. Thus, the main goal of internship courses during a nursing education program should be the development of self-efficacy and providing the highest level of education. Bandura's Self-Efficacy Theory is an instrument that can be applied to assess the degree of certainty in the students' clinical skills performance [15]. According to Bandura's theory, people with sufficient self-efficacy are more likely to adapt to the requirements of a particular situation; on the other hand, people with low self-efficacy face serious problems while performing certain activities. Clinical experience and sufficient education are two major factors that form clinical certainty. Students with higher self-efficacy are efficient and self-regulating while facing future challenges [16]. The modern world requires self-regulating learners. The construct of self-efficacy is crucial in educational environments since Bandura's theory explains that such places fertile lands for growing and forming self-efficacy [17]. The findings of many studies indicate that self-efficacy is quite effective in acquiring knowledge, growing and improving skills, and implementing knowledge and scientific/professional skills [18]. Due to the breakout of the COVID-19 pandemic and the importance of simulation in educating nursing students, the current study was conducted to investigate the effects of simulation-based education and the traditional method on the nursing students' self-efficacy and clinical performance in Kermanshah University of Medical Sciences in 2021.

## Method

### Research design and setting

The current study is quasi-experimental with a pre-test & post-test design. The population included all nursing students in the School of Nursing of Kermanshah University of Medical Sciences, Iran.

#### Sampling

The students were selected according to the simple random sampling technique using the software for producing random numbers (N = 100); then, the first half (50 students) were placed in the control group, while the second group was called the experimental group. The entry criteria included a tendency to take part, conscious consent, and taking internship and training courses. The population included 122 students of nursing doing their Bachelor's degrees, though 22 students left the population due to their lack of interest. First, the objectives of the study were explained to the participants. The students took part in the study with complete consent and agreement. They were informed that whether to participate or not would make any difference in their education.

There were 50 students in the traditional class. The relevant teacher did it, and the educational objectives were based on the lesson plan. The teacher had the necessary professional qualifications due to his teaching experience.

#### The procedures of the experimental group

The participants were divided into an experimental and a control group, and the former group members were educated via the simulation-based approach. The participants' self-efficacy skills and clinical performance before and after the educational programs were evaluated by distributing several questionnaires. At the beginning of the term, the 7^th^ term nursing students in Kermanshah University of Medical Sciences taking the "Surgery" course were divided into an experimental and a control group.

First, the participants of both groups were taken to the practice room to practice items like injection, pneumonia, septic shock, hyperglycemia, and heart massage on a modular according to a checklist. Both groups were filmed, and the pre-test ended without any feedback. Then, the control group received education in the form of PowerPoint presentations and speeches, and some parts of education were presented virtually due to the COVID-19 pandemic. The participants of the experimental group first watched a film, and they received feedback on their errors. The professor taught the course topics like different types of injections on a modular, and the film was handed over to the students. During the next session, the students practiced the topics presented previously on a modular, and the professor presented new topics. One week after the investigation was over, both groups were invited again to gather in the practice room. Then, a test manual was distributed to both groups. Evaluations were conducted by professors unaware of the specific characteristics of the two groups, and scores were given based on the checklist. A single instructor taught the course for 5 consecutive days; each session lasted 2 h.

#### The procedures of the control group

The control group participants were educated via the traditional method (assisted by PowerPoint presentations and speeches) and syllabus, and no specific intervention or change was applied in their educational process. First, the participants took the pre-test consisting of the Questionnaire of Self-Efficacy and an evaluation of their performance.

#### Data collection instrument

A questionnaire of demographic information was distributed to collect information regarding the participant's age and gender. Moreover, the Questionnaire of Nurses' Clinical Self-Efficacy (Charaghi et al., 2010) was distributed. The questionnaire has been developed in Iran and includes 37 items in 5 areas (examining a patient, nursing diagnoses, planning, implementing healthcare programs, and evaluating the healthcare programs) in the form of a 5-point Likert scale. The answers ranged from completely disagree to completely agree, and the scores ranged from 37 to 185. The content and face validity of the questionnaire were approved by a panel of experts consisting of the faculty members of the School of Nursing, and the necessary modifications were applied. The concurrent validity of the "self-efficacy of clinical performance" and "general self-efficacy" instruments was found to be acceptable (*r* = 0.73, *p* < 0.01) [15]. The Cronbach-Alpha coefficient of the finalized instrument "self-efficacy of clinical performance" was α = 0.96 at the range of 0.90–0.92. In addition, another test that was repeated 2 weeks later indicated the convenient stability of the instrument (*r* = 0.94). 

Moreover, the Cronbach-Alpha coefficient of the finalized instrument, "the clinical performance of nursing' was α = 0.72 and a repeated test indicated its stability (*r* = 0.81) [15]. In addition, the instrument's construct validity was confirmed via performing the factor analysis. The clinical performance test was given as pre-defined scenarios to investigate clinical performance. The scenarios were designed and confirmed by the professors of the Department of Nursing and the professor conducting the educational program. Furthermore, the checklist to evaluate the student's performance according to the pre-defined scenarios was developed jointly by the Department of Nursing and the professor.

## Data analysis

The collected data were analyzed using descriptive (mean and SD) and inferential statistical techniques (independent-samples and paired-samples t-test and the COVARIANCE analysis) by SPSS 23.

### Ethical considerations

The research council of Shiraz University of Medical Sciences ratified the current study and was approved and registered by the Committee for Ethics in Shiraz University of Medical Sciences by the ethical code IR.SUMS.REC.1400.287.

### Findings

The average age in the experimental group was 21.84 and the SD was ± 0.570. On the other hand, the average and SD of the control group were 22.2 and ± 0.704, respectively. In addition, 56% of the participants in the experimental group consisted of girls and 44% were boys. On the other hand, 52% of the participants in the control group were girls and 48% were boys.

The obtained results (Table 1) indicated that the control group's mean scores regarding the nurses' clinical self-efficacy were 88.3 and 90.40 in the pre-test and post-test sessions, respectively. Moreover, the normality test performed on the significant values obtained for the research variables (*p* < 0.05) concluded that the data were normal. Thus, parametric tests could test the variables (0.318, 0.612).

**Table 1:** A comparison of the mean and SD of the pre-test and post-test scores obtained by the two groups in terms of the nurses’ clinical self-efficacy

Variable	group	Pretest		Posttest	
		Mean	SD	Mean	SD
**Clinical Self-Efficacy**	Control	88.3	3.078	90.40	3.96
	Experimental	87.58	4.152	142.13	4.37

According to Table 2, the mean scores of the participants' communications before the interventions were 2.16 and 2.14 for the experimental and control groups, respectively. The mean scores for personal management before any intervention were 2.08 and 2.15 for the experimental and control groups, respectively. Besides, the mean scores of being patient-oriented before any intervention were 2.22 and 2.24 in the experimental and control groups, respectively. Moreover, the mean scores of self-description and physical examination before any intervention were 2.18 and 2.20 for the experimental and control groups, respectively. The total pre-test scores of the experimental and control groups in terms of clinical performance were 2.16 and 2.18, respectively. The findings indicated that no significant differences existed between the experimental control groups regarding the four components of clinical performance (communication, personal management, patient-oriented, and self-description and physical examination). The two groups had almost similar mean scores.

**Table 2:** The mean scores of the participants’ clinical performance before performing interventions in the two groups

		**Mean**	**SD**	**Difference**	**t**	***p*-value**	**test**
**Communication**	Experimental	2.16	+ 0.82	0.4	1.94	0.128	T Test
	Control	2.14	+ 0.91				
**Individual management**	Experimental	2.08	+ 0.63	0.7	2.26	0.211	
	Control	2.15	+ 0.74				
**Patient-centered**	Experimental	2.22	+ 0.66	0.02	1.14	0.134	
	Control	2.24	+ 0.84				
**History and physical examination**	Experimental	2.18	+ 0.59	0.02	1.29	0.251	
	Control	2.20	+ 0.62				

According to Table 3, the mean pre-test scores of students in terms of examining their patients were 16.18 in the experimental group and 16.47 in the control group. The mean pre-test scores of nursing diagnoses were 18.32 and 18.60 for the experimental and control groups. Moreover, the mean pre-test scores of the participants in terms of planning were 16.05 in the experimental group and 16.19 in the control group. The mean pre-test scores of the students in terms of evaluating the healthcare program were 17.29 and 17.48 in the experimental and control groups, respectively. Besides, the mean pre-test scores of the students in terms of performing the healthcare program were 19.74 in the experimental group and 19.5 in the control group. The findings indicated that there were no significant differences between the two groups in terms of their mean scores in the 5 components of self-efficacy skills (patient investigation, nursing diagnoses, planning, performing healthcare program, and evaluating the healthcare program), and the two groups showed almost similar mean scores.

**Table 3:** The mean scores of the nursing students’ self-efficacy skills before any intervention in the two groups

		**Mean**	**SD**	**Difference**	**t**	***p*-value**	**test**
**Patient examination**	Experimental	16.18	+ 0.54	0.8	1.18	0.125	T Test
	Control	16.47	+ 0.39				
**Nursing diagnoses**	Experimental	18.32	+ 0.39	0.11	1.30	0.224	
	Control	18.60	+ 0.41				
**planning**	Experimental	16.05	+ 0.68	0.14	2.07	0.183	
	Control	16.19	+ 0.59				
**Implement a care program**	Experimental	19.74	+ 0.34	0.2	1.55	0.506	
	Control	19.56	+ 0.40				
**Care plan evaluation**	Experimental	17.29	+ 0.37	0.2	1.13	0.319	
	Control	17.48	+ 0.42				

In addition, the mean post-test scores of clinical performance obtained by the experimental (educated via the simulation technique) and control groups were 4.57 and 2.24, respectively. Moreover, the SD of the experimental and control groups were 1.96 and 1.37, respectively. Besides, the SEM of the experimental and control groups were 0.3285 and 0.6814, respectively. The significance level of the test pointed to a significant difference between the two groups in terms of their post-test scores.

In addition, the mean post-test scores of the two groups in terms of clinical performance differed significantly (*p* = 0.0001) (the experimental group 4.57 and the control group 2.24). However, no significant difference was observed in their pre-test scores, and the two groups had almost identical mean scores (*p* = 0.211) (the experimental group 2.16 and the control group 2.18).

The results of the COVARIANCE analysis portrayed in Table 4, showed that there a significant difference existed between the mean scores of clinical performance of the experimental group receiving the simulation-based education) and the control group (*P* < 0.01, F (89.27)). Its square was 0.55 meaning that 55% of the variance of clinical performance is explained by simulation-based education. In other words, it was shown that simulation-based techniques affected the nurses' clinical performance.

**Table 4:** The results of the COVARIANCE analysis of the two groups in terms of their clinical performance

Performance clinical	T.S	df	M.s	F	sig	Effect level	*p*-value
Pretest	89.27	98	98.27	13.76	0.001	0.55	0.05
Posttest	158.13	1	51.59	29.29	0.01	0.06	
Error	345.24	38	9.16				

Comparing the nursing students' mean pre-test and post-test scores of self-efficacy skills in terms of the simulation-based technique in the experimental and control groups.

It can be observed in Table 5, that the mean post-test scores obtained by two groups in terms of self-efficacy skills differed significantly (*p* = 0.0001); however, no significant difference was observed in the two groups' pre-test scores, and they had almost similar mean scores (*p* = 0.384).

**Table 5:** The paired-samples t-test to investigate the difference between the participants’ pre-test and post-test scores in terms of their self-efficacy skills

	Variable		Mean	SD	T	*P*-Value
Pretest	Self-efficacy skills	Experimental	87.57	.39418	5.92	0.384
		Control	88.3	0.42502		
Posttest	Self-efficacy skills	Experimental	142.13	0.58354	6.39	0.001
		Control	90.40	0.47120		

The results of the COVARIANCE analysis in Table 6, show that a significant difference existed between the self-efficacy skills of the experimental and control groups (*p* < 0.01, F (20.83)). Its square was 0.58, meaning that 58% of the self-efficacy skills were related to simulation-based learning. In other words, it can be argued that the simulation-based technique significantly impacted the nursing students' self-efficacy skills.

**Table 6:** The results of the COVARIANCE analysis of the groups in terms of the level of self-efficacy skills

Self-efficacy skills	Test	T.S	df	M.s	f	Sig	Effect level	*p*-value
Pretest (Experimental)		13.57	1	42.15	20.83	0.001	0.58	0.05
Posttest (Control)		13.35	39	14.69	8.66	0.1	0.04	-
Group		7.401	1	7.401	36.17	0.000	-	0.01
Error		26.93	20	48.13	-	-	-	-
Total		251.83	38	-	-	-		-

## Discussion

The findings indicated that the nursing students' mean scores in terms of self-efficacy skills increased after the intervention (142.13) compared to their pre-test scores (87.57). In addition, simulation-based education was influential on the students' self-efficacy skills. Besides, the students' mean clinical performance scores increased after the intervention (4.57) compared to their pre-test scores (2.16); thus, it was shown that the simulation-based technique influenced the nursing students' clinical performance. In justifying the obtained results, it can be argued that implementing the simulation-based technique in medicine makes it possible for the students to face rare occasions and intervene in them. It improves group work and the related skills in the would-be doctors and increases the group members' self-confidence [19]. The current study's findings were in line with Sajjadi et al., Kargar et al., and Rahmani et al., [11, 18, 20]. Sajjadi et al. showed that the participants educated with an online simulation-based technique received higher scores in terms of satisfaction after the intervention and 10 days later reported better scores in terms of their performance compared to the speech-based group. Moreover, it was shown that 10 days after the intervention, the time taken to execute a certain scenario in the online simulation-based group was significantly lower than the speech-based group [11]. Moreover, Kargar et al. conducted a study titled "a study on the effects of simulation on nurses' performance in neonatal resuscitation in Behbahani Hospital" ad showed that the level of knowledge in the control and experimental groups increased by 9 and 10 scores, respectively; thus, the difference between the two groups was not significant. In addition, no difference was observed between the two groups regarding the pre-test performance. However, a significant difference was observed one month after the educational program, and the participants in the experimental group performed significantly better than their counterparts in the control group. The findings indicated that simulating and simultaneously filming for educational purposes is more effective on learning [18]. Heydarzadeh et al. conducted a study titled "the effects of computerized simulation and implementing models on the nursing students' perception of self-efficacy during cardiopulmonary resuscitation". The mean scores and the SD of the students' self-efficacy perceptions before and after the intervention in both groups were significantly different (23.5 in the model-based group and 15.4 in the computerized simulation group). The study's findings showed that either of the above techniques increased self-efficacy perceptions concerning cardiopulmonary resuscitation; thus, educational institutions can implement either technique for educating their students depending on their particular situations and facilities and train nurses with higher qualifications to provide service for their patients [21]. Implementing modern educational techniques such as simulators and educational films can effectively increase students' cognitive, communicative, and practical skills [22], which was in line with the findings of the current study. A study by Khalaila indicated that implementing the simulation-based technique increased the mean scores of nurses' self-efficacy after an intervention program [23]. Liaw et al. implemented the simulation-based technique in control and experimental groups. The experimental group (including 15 people) started a 6-h simulation program providing 4 clinical scenarios concerning pneumonia, shocks, hyperglycemia, and septic shock to manage a critical patient, and a question and answer session was held at the end. Moreover, a performance assessment was performed using a videotape and a panel of assessors who were unaware of the study's objectives. Comparing the pre-test mean scores did not indicate any significant differences between the two groups, but a significant difference was observed in the post-test performance of the experimental group [24]. The study by Rodrigues et al. indicated that implementing simulation-based educational software is a useful strategy to assist nurses and develop their professional skills and training [25]. The study by Fidalgo et al. on pharmacy students showed that implementing films, moving pictures, designs, and texts in integrated content can significantly enhance the students' conceptual understanding and learning [26]. Moreover, the study by Lis et al. on a group of medical students indicated that implementing educational films in the experimental group was useful in improving the students' self-efficacy and clinical skills [27]. The findings were found to be in line with the findings of the current study. Thus, people's understanding of their duties and the management of critical conditions, their belief in being able to show a successful performance, and presenting accurate solutions to attain desirable consequences are clear representations of self-efficacy. If they are well-represented in nurses, their clinical performance and the quality of their healthcare activities will be enhanced. Lee et al. conducted a study on nurses and indicated that simulation-based education has significant effects on the nurses' qualifications and clinical performance  [28], which was in line with the findings of the current study. Furthermore, the findings of Hsin-Hsin Lin showed that the simulation-based approach significantly impacts nursing students' self-efficacy and performance qualifications [29], which was also in line with the findings obtained in the current study. Yon Hee Seo et al. showed that simulation-based education significantly impacts Korean nursing students' clinical argument, problem-solving processes, self-efficacy, and clinical qualification [30], which was in line with the current study's findings. Moreover, the findings of Salwa et al. indicated that the simulation-based approach significantly impacts nursing students' communicative skills, self-efficacy, and clinical qualification [31]. 

Bandura (1997) has stated that people with high self-efficacy beliefs show more resistance when dealing with problems; According to Bandura, self-efficacy encourages motivation and cognitive resources of a person and is a factor for exercising control over a specific event. According to Bandura, a person's emotions, thinking, and behavior in any situation depend on his sense of ability [32]. In situations where a person is confident about his/her abilities, his behavior, cognition, and emotions are entirely different from situations where a person feels incompetent and insecure or lacks competence.

### Limitations

The current study was just conducted on the students of nursing. Thus, generalizing the findings to other fields of medical sciences is somewhat troublesome.

## Conclusion

Simulation as a method of education consists of all activities that mock a real-world clinical environment and has been designed to illustrate processes, decision-making, and critical thinking by role-playing and instruments like educational films and models. Simulation-based education makes it possible to transmit the knowledge and skills required for the healthcare activities performed by a nurse, and it can enhance students' evaluation of their capabilities.

## Data Availability

The datasets analyzed during the current study are available from the corresponding author upon reasonable request.
